# The dilemma of managing thyroid gland after incidental diagnosis of malignant struma Ovarii. Is radical thyroidectomy and radioactive iodine Necessary? A case report and literature review

**DOI:** 10.1016/j.gore.2023.101189

**Published:** 2023-04-17

**Authors:** Wiktoria Irena Batog, Ciarán Ó Riain, Feras Abu Saadeh

**Affiliations:** a Trinity College Dublin, Dublin 2, Ireland; b Department of Gynecology, St. James’s Hospital, Dublin 8, Ireland; c Department of Histopathology, St. James’s Hospital, Dublin 8, Ireland; d Trinity St. James’s Cancer Institute, Dublin 8, Ireland

## Abstract

• Struma ovarii is a rare ovarian teratoma which consists of at least 50% thyroid tissue.
• In 0.5–1% of cases the thyroid tissue in the struma ovarii undergoes a malignant transformation.
• Patients should be divided into low and high-risk groups for synchronous thyroid carcinoma.
• Patients with abnormalities on thyroid imaging should be considered as high-risk.
• A thyroidectomy and radioactive iodine treatment can decrease the risk of recurrence.

## 1 Introduction

Mature cystic teratoma is a common ovarian germ cell tumor comprising 10–20 % of all ovarian germ cell tumors (Pradhan and Thapa, 2014). 80% of mature cystic teratomas occur during reproductive age. Although Mature cystic teratomas are relatively common, malignant transformation occurs in only 1–3% of cases (Ulker et al., 2012). Squamous cell carcinomas account for 80% of secondary malignant transformations of ovarian teratomas, the remaining mature cystic teratomas are carcinoid tumors, adenocarcinomas, and thyroid carcinomas (Qin et al., 2021).

Struma ovarii a designation given to an ovarian mature teratoma with predominant or sole component of thyroid tissue. Malignancy within struma ovarii occurs in 0.5–1%. (Qin et al., 2021). It can be classified into three types: papillary, follicular variant of papillary and follicular (Hinshaw et al., 2012). Due to the rarity of malignant struma ovarii (MSO), the management of thyroid gland remains undecided and controversial. Metastasis and recurrence rates are low (Salman et al., 2010; 2010:).

## 2 Case presentation

We present a case of 48-year-old women, a mother of three children, two vaginal delivery and one caesarean section. She had a normal cycle and used the oral contraceptive pill for four years. She was an ex-smoker and had a moderate alcohol consumption. Her medical history is limited to Crohn’s disease for which she was treated with azathioprine and mesalamine.

**Fig. 1:**
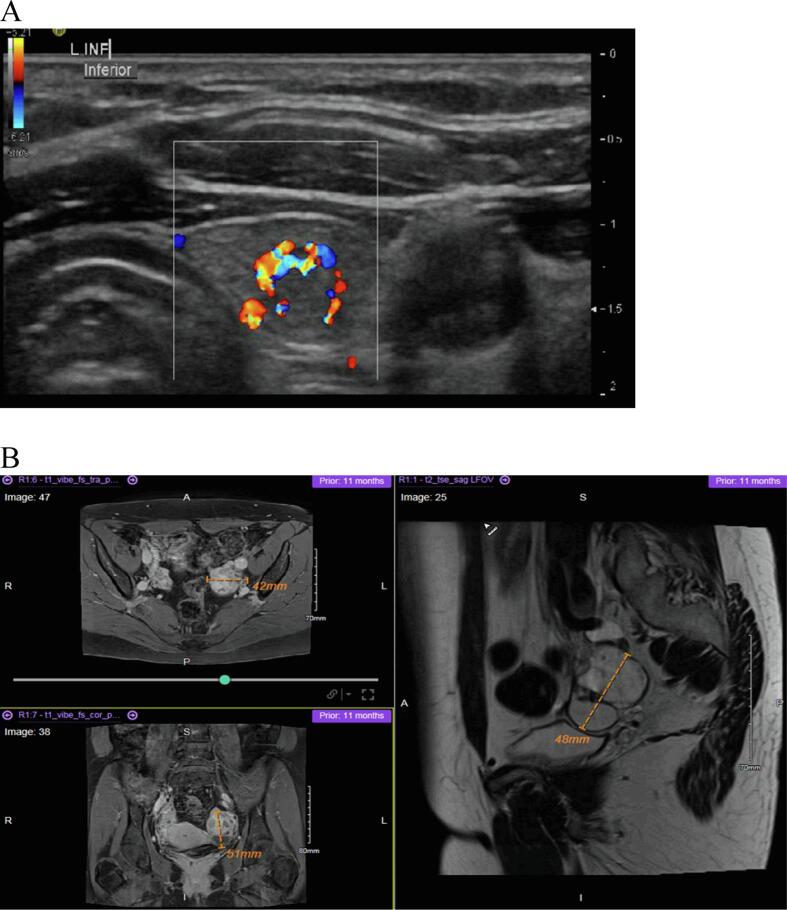
A: Ultrasound image showing a strong doppler blood flow in the ovary (single column width). B: Pelvic MRI with contrast showing a 4.5cm x 2.5cm x 5.0cm T2 hyperintense multiseptated left adnexal mass (full two-column width).

The patient had no symptoms; she was enrolled in a clinical trial for Crohn’s disease. On routine image screening (as part of the trial protocol) a complex left ovary lesion was identified. The cyst presented with nodularity and a strong doppler blood flow (Fig. 1-A). The patient underwent a pelvic MRI with contrast. A complex 4.5 cm × 2.5 × 5.0 cm encapsulated predominantly T2 hyperintense multiseptated left adnexal mass was identified. There was no internal fat to suggest a dermoid, no T1 hyperintensity to indicate endometrioma and no T2 hyposensitivity to suggest fibroma (Fig. 1-B). Tumor markers were normal.

The patient’s case was discussed at the gynecology cancer multidisciplinary meeting. In view of the unusual finding on MRI and patient wished to maintain fertility, hence the multidisciplinary meeting recommendation was for fertility sparing surgery. The patient underwent a laparoscopic staging surgery that included peritoneal washing, unilateral salpingo-oophorectomy, omentectomy, appendicectomy, peritoneal biopsy as well as a left pelvic and *para*-aortic lymph node dissection. The cyst was delivered in a bag through an umbilical port, however the cyst leaked during manipulation.

**Fig. 2:**
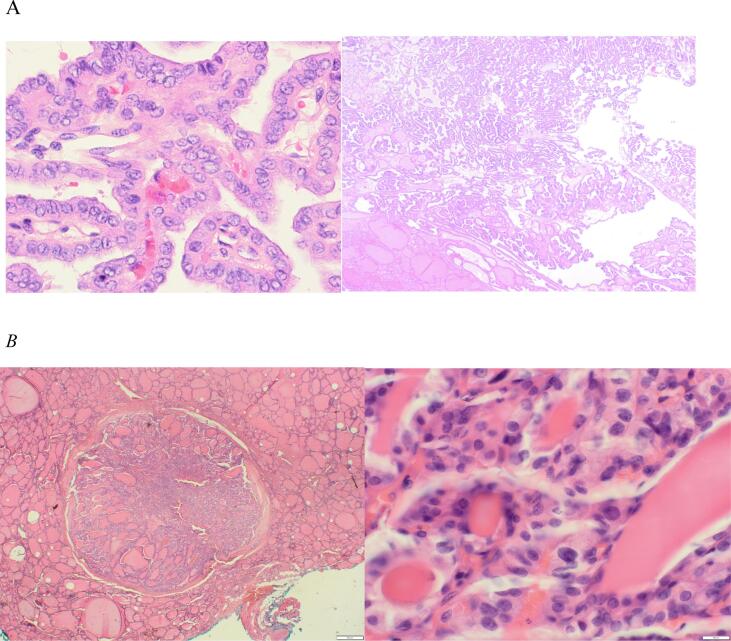
A: Histopathology slide of the ovary. Low power view of neoplasm with papillary architecture. Residual normal thyroid follicles filled with colloid present at bottom left. (single columns width). B: Histopathology slide of the thyroid showing a circumscribed follicular pattern above and irregular nuclear membranes and grooves below (Single columns width).

Peritoneal fluid cytology reported no malignant cells. Histopathological interpretation of ovary was of papillary thyroid carcinoma (25 mm) arising within left ovarian struma ovarii. The tumor's FIGO stage was IC, by the virtue of cyst rupture. There was a focal lymphovascular space invasion (Fig. 2-A). The tumor was appropriately positive for thyroid markers TTF-1 and thyroglobulin, and negative for neuroendocrine markers; chromogranin and synaptophysin. The peritoneal and omental biopsy, appendix, left common iliac lymph nodes and left *para*-aortic lymph nodes were negative for malignancy. There were no intra or post operative complications.

**Fig. 3:**
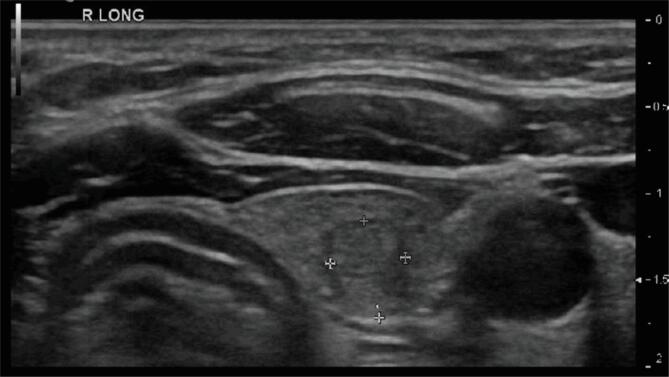
Thyroid Ultrasound showing an ill-defined, low attenuation 5mm nodule in the lower left lobe (single column width).

In view of the unexpected diagnosis of thyroid cancer within the struma ovarii, the case was discussed at the head and neck cancer multi-disciplinary meeting. The patient underwent a CT neck, thorax, abdomen and pelvis with contrast, no metastasis was noted. The thyroid gland was normal in size with symmetry of right and left lobes. It contained an-ill-defined indeterminate low-attenuation 5 mm nodule in the lower pole of the left lobe with no lymphadenopathy (Fig. 3).

The tumor markers (CEA, CA125 and Ca19.9) were negative. The thyroid function tests, and thyroglobulin were within normal limits. An ultrasound guided fine needle aspiration cytology (FNAc) was performed from the thyroid nodule on the lower left lobe. The FNA cytology was consistent with normal thyroid tissue with no evidence of malignancy. Despite the negative cytology the nodule raised suspicion. The case was discussed again at head and neck multidisciplinary meeting and the decision was for a total thyroidectomy. Histopathological interpretation was of a small 6 mm nodule of follicular variant of papillary thyroid carcinoma (Fig. 2-B). The patient had adjuvant radioactive iodine treatment. No further treatment was deemed necessary at that time. At the time of discharge, the patient was prescribed azathioprine and mesalamine for her Crohn’s disease, levothyroxine to treat hypothyroidism and cholecalciferol for vitamin D deficiency.

The patient has been followed up every six months for the past six years. No evidence of recurrence has been noted.

## 3 Discussion

Struma ovarii was first described at the start of the 20th century and describes an ovarian teratoma that consists of at least 50% thyroid tissue. Struma ovarii peaks in the fifth decade of life. There have been reported cases of post-menopausal women, however struma ovarii in prepubertal girls is rare (Roth and Talerman, 2007). It may present with ascites in 17% of cases. Hyperthyroidism occurs in 5–8% of cases. Yoo et al. (Yoo et al., 2008) noted that 41.2% of cases were asymptomatic and tumors were discovered accidentally during routine ultrasound check-up. The tumor can be macroscopically described as a solid or gelatinous, brown, or green mass. Imaging of struma ovarii is non-specific. A classic MRI described by Matsuki et al. includes multiple intracystic areas with low signal intensity on T2 and intermediate signal intensity on T1 (Matsuki et al., 2000). The definitive diagnosis of struma ovarii can only be rendered through histopathological examination of the surgical specimen. The predominant treatment includes a total hysterectomy with bilateral salpingo-oophorectomy, omentectomy, peritoneal washings, and lymph nodes sampling, however a unilateral oophorectomy can be performed to preserve fertility (Russo et al., 2016).

The criteria for malignant struma ovarii are like those used to evaluate primary thyroid carcinomas although follicular neoplasms may be difficult to assess as there may be no capsule to assess for capsular invasion. The use of immunohistochemistry such as Cytokeratin 19, HBME-1, and galectin-3 can aid the differentiation between benign thyroid tissue and papillary carcinoma of the thyroid in the struma ovarii (Salman et al., 2010; 2010:). Papillary struma ovarii may harbor oncogenic activation of BRAF (35% to 69%), RAS (10%), or RET (5% to 30%). These mutations correlate with tumor subtype, patient age, and clinical behavior (Schmidt et al., 2007).

In rare cases the thyroid tissue in the struma ovarii undergoes a malignant transformation (Qin et al., 2021). In their review Siegel et al. reported the incidence to be low, approximately 6% (Siegel et al., 2019). Patients with both malignant struma ovarii and thyroid cancer can be classified into three groups; metastasis of thyroid cancer to the ovary; metastasis of malignant struma ovarii to the thyroid; and concurrent occurrence of thyroid cancer and malignant struma ovarii (Xiao et al., 2022). Ovarian metastasis to the ovary is extremely rare and the ovarian tumors are therefore considered to be independent primaries (Cui et al., 2021). It’s hypothesized that multifocal thyroid tumors arising synchronously in different locations are likely due to a genetic predisposition (Cui et al., 2021).

There’s an ongoing debate on the utilization of radical treatment such as I^131^and a thyroidectomy as treatment methods for a potential synchronous thyroid carcinoma. As a results of its rarity, there are no guidelines regarding the assessment of the thyroid and risk stratification. Hinshaw et al. (Hinshaw et al., 2012) proposed a guide to post-operative treatment of the thyroid. Low risk tumors were defined as confined to the struma ovarii, less than 2 cm with poorly differentiated features. The treatment would be limited to TSH suppression and thyroglobulin measurements. Unfortunately, no criteria were provided for high-risk patients.

Patient assessment for a synchronous thyroid malignancy is advised in all cases of malignant struma ovarii. Thyroglobulin and TSH has been previously reported as a sensitive tumor marker used for clinical monitoring of thyroid carcinoma and malignant struma ovarii (Cui et al., 2021, DeSimone et al., 2003), however it’s efficacy in the prediction of patients that are at a high risk for a synchronous thyroid carcinoma is debatable. In their literature review Salaman et al. suggested that thyroglobulin and serial I^131^ whole body could help to guide the decision regarding further management of the thyroid. Since 98% of thyroid cancers with serum thyroglobulin > 10 ng/ml are clinically disease free, they proposed that only patients with serum thyroglobulin > 10 ng/ml should receive I^131^ (Salman et al., 2010; 2010:). Contrastingly, Russo’s et al. patient exhibited subclinical hyperthyroidism; however, their thyroglobulin levels were within range. Thyroid ultrasound depicted chronic thyroiditis therefore the patient underwent a thyroidectomy alongside the removal of the primary tumor (Russo et al., 2016). Siegel’s et al. patient was deemed as clinically euthyroid, meanwhile papillary thyroid carcinoma was reported on FNA, hence the patient underwent a thyroidectomy (Siegel et al., 2019). Similarly, our patient’s thyroglobulin levels, and thyroid function tests were within normal range, despite the presence of papillary thyroid carcinoma. The involvement of endocrinology and surgical oncology on the case and biochemical investigations can serve as a method of risk stratification, nonetheless the presence thyroid carcinoma can’t be excluded on basis of thyroglobulin and TSH levels.

Recurrence rates in patients who don’t undergo I^131^ or a thyroidectomy have been highlighted in DeSimone’s study (DeSimone et al., 2003). The series reviews 24 patients with malignant struma ovarii; 16 received no treatment for their thyroid and were followed up clinically with or without their serum thyroglobulin used as a marker for potential recurrence, hence the management of their thyroid was conservative. The remaining 8 patients received treatment. There were 8 cases of recurrence, occurred in the group of 16 patients who were managed conservatively by clinical review only. There were no recurrences in the women who were treated postoperatively with adjunctive therapy who had an initial complete response. Taking into consideration the above results the authors of that study believe that I^131^ should be considered in the first-line treatment for malignant struma ovarii after surgical diagnosis and treatment. This treatment should be preceded by a thyroidectomy which serves a dual function; firstly, it allows for a pathological assessment of the thyroid, secondly it potentiates the effect of I^131^ therapy (DeSimone et al., 2003). Contrastingly Cui et al. found that the mortality in patients who received I^131^ was decreased by 1.5% and was insignificant (Cui et al., 2021). In the literature recurrence rates range from 7.5 to 38% (Siegel et al., 2019).

In our case the patient underwent a removal of the ovarian mass. Previous literature suggested the presence of a concurrent thyroid carcinoma with stuma ovarii, therefore the patient was investigated for residual cancer with CT of neck, thorax, abdomen, and pelvis, which depicted a nodule in the lower left pole of the thyroid. The patient’s thyroglobulin levels were unremarkable. The presence of the nodule was confirmed by ultrasound and FNAc reported no malignancy in the thyroid. Despite negative cytology and normal thyroglobulin levels, the presence of a nodule was alarming. A decision to perform a thyroidectomy was made, and it demonstrated follicular variant of papillary thyroid carcinoma. In some cases, like ours the presence of thyroid carcinoma concurrent with malignant struma ovarii may not be obvious.

There’s a need for a risk stratification algorithm, which could be based on that proposed by Hinshaw (Hinshaw et al., 2012). When the thyroid surveillance depicts no abnormalities, there’s no strong evidence to advocate for a thyroidectomy and I^131^ treatment, hence the patient can be classified as low risk. Peculiarities on thyroid imaging should be a defining feature of a high-risk group, even in the absence of biochemical or cytological abnormalities These patients should proceed with a thyroidectomy and I^131^. Long-term follow-up and multidisciplinary input including endocrinology and surgical oncology is recommended in all cases.

## 4 Conclusion

Malignant struma ovarii is an infrequent phenomenon. Moreover, the preoperative diagnosis of malignant struma ovarii and concurrent thyroid carcinoma is challenging. A histopathological examination is the gold standard for diagnosis in both cases. Thyroidectomy and I^131^ treatment are controversial especially in absence of obvious biochemical or cytological disease. Ideally a risk stratification system dividing patients into low and high-risk groups should be established. Case series like our case, did show that a radical treatment of thyroid with surgery and I^131^ in the presence of abnormalities on thyroid imaging may decrease recurrence and improve survival.

## Informed Consent Statement

Written informed consent was obtained from the patient for publication of this case report and accompanying images. A copy of the written consent is available for review by the Editor-in-Chief of this journal on request.

## CRediT authorship contribution statement

**Wiktoria Irena Batog:** Investigation, Writing – original draft, Writing – review & editing, Visualization. **Ciarán Ó Riain:** Resources, Writing – review & editing. **Feras Abu Saadeh:** Conceptualization, Resources, Supervision, Project administration, Writing – review & editing.

## Declaration of Competing Interest

The authors declare that they have no known competing financial interests or personal relationships that could have appeared to influence the work reported in this paper.
